# Complete atrioventricular block under epidural ropivacaine infusion in a patient with first-degree atrioventricular block and myasthenia gravis: a case report

**DOI:** 10.1186/s40981-022-00524-5

**Published:** 2022-04-28

**Authors:** Takuma Hashimoto, Shinobu Kuratomi, Hayashi Yoshimura

**Affiliations:** Department of Anesthesiology, Saiseikai Fukuoka General Hospital, Tenjin 1-3-46, Chuo-ku, Fukuoka-shi, Fukuoka-ken 810-0001 Japan

**Keywords:** Epidural anesthesia, Arrhythmia, Complete atrioventricular block, Myasthenia gravis

## Abstract

**Background:**

First-degree atrioventricular block (AVB) may lead to complete AVB. Herein, we present a case of a complete AVB under thoracic epidural catheter infusion of ropivacaine with fentanyl in a patient with first-degree AVB and myasthenia gravis.

**Case presentation:**

A 74-year-old woman with first-degree AVB underwent thymectomy for myasthenia gravis. Continuous thoracic epidural catheter infusion of 0.2% ropivacaine with fentanyl was initiated at 15 min before the end of the surgery. At 9 h postoperatively, the electrocardiogram showed a 10-s-long pause due to complete AVB. Thus, a temporary pacemaker was implanted, and at 19 h postoperatively on postoperative day 1, cardiac pacing was initiated and lasted approximately 30 s. After catheter removal, she had no further episodes of complete AVB.

**Conclusion:**

First-degree AVB may lead to complete AVB under the influence of thoracic epidural infusion of ropivacaine in patients with myasthenia gravis.

## Background

Complete atrioventricular block (AVB) is a rare occurrence that may be triggered by surgical vagal and non-surgical stimuli in addition to stimuli from myocardial ischemia and cardiac conduction anomalies [1].

However, whether epidural anesthesia triggers complete AVB or not is not clearly indicated. To our knowledge, there has been only one case of complete AVB caused by epidural anesthesia alone, which was the first reported case in Japan [2].

Hence, we present a case in which epidural ropivacaine infusion induced complete AVB in a patient with myasthenia gravis (MG) and first-degree AVB.

## Case presentation

**Fig. 1:**
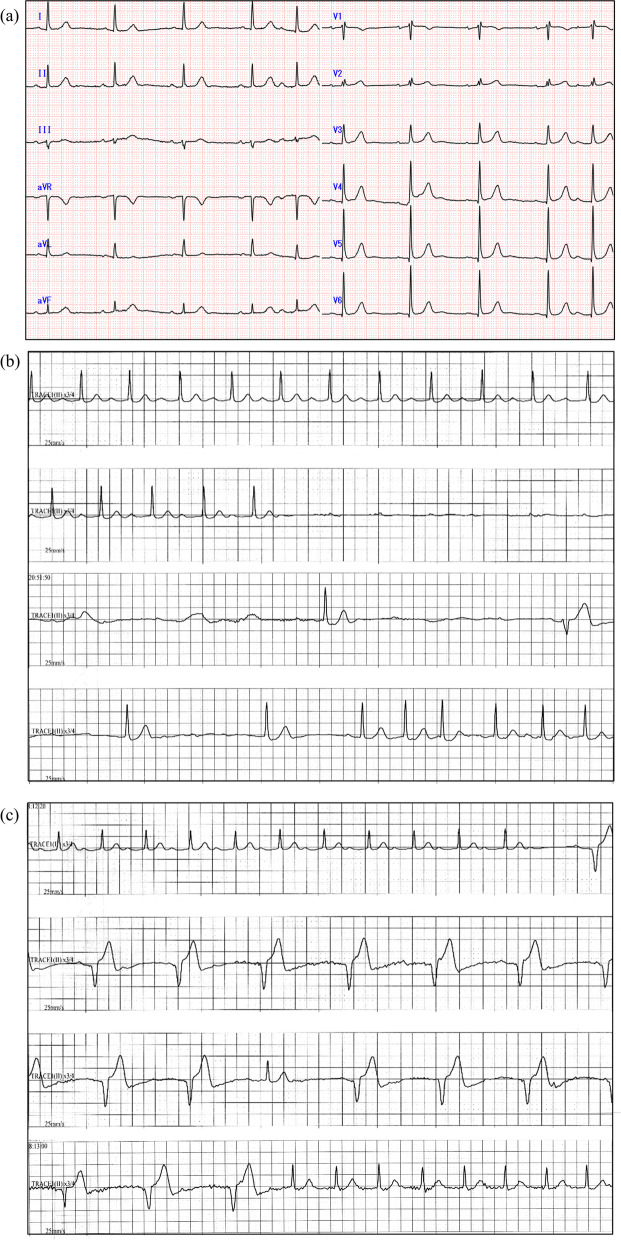
Electrocardiogram before anesthesia (**a**), at 9 h postoperatively in II lead (**b**), and after implantation of a temporary pacemaker in II lead (**c**). **a** First-degree atrioventricular block (AVB). **b** Complete AVB lasted for 10 s (and recovered without treatment). **c** Pacemaker-generated waves (40 per min) after the absence of QRS complex for 4 s.

A 74-year-old woman (height, 156 cm; weight, 58 kg) was scheduled to undergo thoracoscopic thymectomy for MG associated with thymoma. The symptoms of MG included ptosis and muscle weakness, for which at 1.5 months preoperatively, she was administered a 1-week course of ambenonium and methylprednisolone pulse therapy that markedly improved the symptoms by the time of the surgery. She also had a history of tachyarrhythmia; however, on initiating treatment with metoprolol, the palpitations subsided and no further episodes of bradycardia or syncope occurred. Dyslipidemia was another comorbidity. Her blood test results were positive for thyroglobulin, acetylcholine receptor, and thyroid peroxidase antibodies, but no electrolyte imbalance was noted. Preoperative electrocardiogram (ECG) showed a first-degree AVB (PR interval of 0.204 s) (Fig. 1a). Transthoracic echocardiography showed mild to moderate aortic regurgitation and mitral regurgitation, but the ejection fraction was preserved.

The surgery was performed under combined general and epidural anesthesia. An epidural catheter was inserted at the T5–6 interspace. No bradycardia was observed during the procedure or at the time of administration of the test dose. In addition to the standard parameters, including blood pressure, pulse oximetry, and ECG, invasive blood pressure was also monitored. Neuromuscular blockade was monitored via visual observation of the train-of-four count. Anesthesia was induced via intravenous administration of fentanyl, propofol, and rocuronium, and maintained using desflurane (EtDes, 3–5%) and remifentanil (0.2–0.3 mcg/mg/min). At 15 min before the end of the surgery, thoracic epidural catheter infusion of 0.2% ropivacaine and fentanyl was initiated with an initial dose of 5 mL. No bradycardia or other arrhythmias were noted during the surgery that lasted for 3 h 52 min. The train-of-four count throughout the surgery was 0–1. Extubation was performed without difficulty. However, she complained of chills and started shivering; therefore, pethidine (35 mg) was administered. Although she was mildly sedated after pethidine administration, she did not complain of pain, and her respiratory status was stable. Therefore, she was returned to the intensive care unit after adequate observation.

At 2 h postoperatively, she was awake and had no complaints of pain. However, her heart rate (HR) dropped to 15 bpm, and she suddenly became nauseous at 5 h postoperatively. After vomiting, her HR improved, and she did not lose consciousness during this episode of bradycardia. At 9 h postoperatively, severe bradycardia for 22 s with a 10-s-long pause due to complete AVB was observed on the ECG (Fig. 1b). After the event, 12-lead ECG was performed, but no complete AVB was detected. Moreover, transthoracic echocardiography did not reveal any wall motion abnormalities. Her high-sensitivity troponin T level was slightly elevated at 0.042 ng/mL, but no electrolyte imbalance was noted.

Based on the previously mentioned symptoms and the ECG, our cardiologist diagnosed complete AVB, and implanted a temporary pacemaker programmed to VVI 40 via the right jugular vein. Subsequently, emergency coronary angiography was performed to investigate ischemic heart disease, but no significant stenosis was observed in the coronary arteries. During coronary angiography, her HR dropped with nausea and cardiac pacing started, but returned to sinus rhythm within a few seconds.

At 19 h postoperatively on postoperative day 1, her HR dropped again with nausea and cardiac pacing started that lasted approximately 30 s (Fig. 1c). Thoracic epidural administration was discontinued at 21 h postoperatively because of its adverse effect on her HR. Thereafter and until postoperative day 2, no further complete AVB was observed. On postoperative day 3, atrial fibrillation appeared and the HR increased to 110–170 bpm; therefore, bisoprolol tape administration was initiated. After the initiation of bisoprolol, the HR decreased, and she developed tachycardia-bradycardia syndrome. Because of this syndrome, complete AVB, and after considering her request, we implanted a permanent pacemaker and programmed it to manage the ventricular pacing (MVP™; Medtronic, Minneapolis, MN, USA) mode on postoperative day 8. She was discharged from the hospital on postoperative day 17. Pacemaker checks were performed at 1 and 6 months after the implantation and showed no atrial fibrillation or complete AVB.

## Discussion

In our case, continuous epidural 0.2% ropivacaine with fentanyl infusion likely caused complete AVB in a patient with first-degree AVB with MG. The most likely cause of complete AVB was the sympathetic blockade by thoracic epidural anesthesia (TEA). Other factors, including first-degree AVB, MG, the toxicity of ropivacaine itself, and nausea and vomiting, must be considered.

Human preganglionic sympathetic cardiac neurons originate mainly from thoracic spinal segments T1–T4 or T5 [3]. In the present case, the epidural catheter was placed at the cardiac sympathetic nerve level, and the event disappeared after catheter removal, suggesting that sympathetic suppression by TEA caused complete AVB. Third-degree heart block has been documented following TEA in a patient with first-degree AVB [2]. In dogs, TEA prolonged atrioventricular node conduction time [4]. These reports suggest that local anesthesia administered to the epidural space influences the electrophysiological functions via the sympathetic nervous system.

First-degree AVB was considered an entirely benign condition. In fact, some reports argue that it is a misnomer as there is only delay and no actual block in the AV conduction. However, it has long been acknowledged that extreme forms of first-degree AVB (typically a PR interval exceeding 0.30 s) can cause symptoms because of inadequate timing of atrial and ventricular contractions, similar to the so-called pacemaker syndrome [5]. Our patient had no preoperative symptoms and the PR interval was not extremely long (0.204 s). The patient in the aforementioned report of complete AVB due to TEA also had first-degree AVB [2], which may have increased the risk of complete AVB during epidural anesthesia.

MG is known to involve other body systems including the heart. MG patients have a higher prevalence of cardiac manifestations in the presence of thymoma (10–15%) [6]. Especially, the anti-Kv1.4 antibody was associated with a more severe form of MG, lethal arrhythmias including ventricular tachycardia, sick sinus syndrome, complete AVB, and sudden death [6, 7]. She was not tested for anti-Kv1.4 antibody, but she did not show severe arrhythmia or syncope preoperatively. Therefore, we believe that factors, such as surgery and anesthesia, were the cause of complete AVB, but we cannot rule out the possibility that MG had an effect.

Epidural ropivacaine infusion has caused fluctuating arrhythmia [8], and ropivacaine even under a toxic dose may cause dysrhythmia. In contrast, animal studies have demonstrated that ropivacaine has less potential for cardiotoxicity [9]. The toxicity of ropivacaine inducing arrhythmias cannot be ruled out, as the blood levels of ropivacaine were not measured. In the present case, the dose of ropivacaine she had received until reaching complete AVB (i.e., 60 mg over 5 h and 200 mg over 19 h) was below the Japanese maximal recommended doses listed in package insert.

She frequently experienced nausea and vomiting during the bradycardia attacks, consistent with the symptoms caused by decreased cerebral blood flow. However, we cannot deny the possibility that nausea and vomiting may have stimulated the vagal afferent pathway, thus, resulting in bradycardia caused by parasympathetic excitation. Either the increased intrathoracic pressure during retching or the increased esophageal pressure during the vomiting episodes could have elicited a vagal response [10]. A balloon dilation actually causing AVB with reproducibility has been previously reported [11]. However, as the patient had experienced no previous episode of syncope accompanied by nausea and vomiting, we concluded that it would have been difficult for vomiting to have caused complete AVB.

This report had some limitations. We did not measure anti-Kv1.4 antibody and the blood levels of ropivacaine. If these were measured, there would have been more evidence on their impact on complete AVB. In conclusion, the risk of complete AVB should always be considered when administering epidural ropivacaine, even in asymptomatic first-degree AVB patients. Patients, especially those with MG, should be monitored accordingly.

## Data Availability

Not applicable.

## Abbreviations

AVB: Atrioventricular block
MG: Myasthenia gravis
ECG: Electrocardiography
HR: Heart rate
TEA: Thoracic epidural anesthesia
